# Association of triglyceride glucose index with all-cause and cause-specific mortality among middle age and elderly US population

**DOI:** 10.1186/s12877-022-03155-8

**Published:** 2022-05-28

**Authors:** Min Sun, Hongmei Guo, Yi Wang, Dongchao Ma

**Affiliations:** Institute of Hypertension, Jiangsu Province Official Hospital, 30 Luojia Road, Nanjing, 210029 China

**Keywords:** Triglyceride glucose index, All-cause mortality, Cause-specific mortality, Insulin resistance

## Abstract

**Aims:**

To investigate the association between the triglyceride glucose (TyG) index and all-cause and cause-specific mortality in middle age and elderly population.

**Methods and results:**

A total of 9,254 participants with age ≥ 45 years were enrolled from the National Health and Nutrition Examination Survey cycle of 1999–2014. The TyG index was determined as ln [fasting triglycerides (mg/dL) x fasting glucose (mg/dL)/2]. Primary outcomes were all-cause mortality and cause-specific mortality (cardiovascular diseases and malignant neoplasms). The association between the levels of TyG and the risk of mortality was explored with Cox regression models. After a median follow-up of 7.6 years, 1,774 all-cause death occurred. Univariate analysis showed that the TyG was associated with all-cause mortality (hazard ratio [HR] 1.18, 95% confidence interval [CI] [1.11,1.26]; *p* < 0.001). Furthermore, multivariate-adjusted analysis found that the third TyG quartile (8.72 ~ 9.16) was associated with the lowest risk of all-cause mortality (HR 0.84, 95%CI [0.73, 0.98]; *p* < 0.05). Restricted cubic splines showed that the association between levels of TyG index and the risk of all-cause mortality was non-linear (p for nonlinearity < 0.001) and the inflection point was 9.18 using threshold effect analysis. The HR was 0.82 (95%CI [0.71,0.96]) below 9.18 while the HR was 1.32 (95%CI [1.12,1.55]) above 9.18.

**Conclusion:**

TyG index was U-shaped associated with all-cause mortality and the TyG index associated with the lowest risk of all-cause mortality was 9.18.

## Introduction

The triglyceride glucose (TyG) index has been suggested as a surrogate marker of insulin resistance [1, 2]. It was a promising biomarker for glycemic control in diabetic patients and paralleled the prevalence of metabolic syndrome and its components [3, 4].

Several studies have examined the associations between TyG index and all-cause and cause-specific mortality [5, 6]. It has been demonstrated that TyG index was an independent predictor of mortality in patients with type 2 diabetes [7], hypertension [8] and stroke [9]. Besides, the positive relationship was also seen in subclinical myocardial injury [10], ST-elevation myocardial infarction [11] and non-ST elevation acute coronary syndrome [12]. In the healthy population, elevated TyG index was associated with an increased risk of all-cause mortality and cardiovascular mortality [13]. However, it was not clear about the predictive role of TyG index on all-cause and cardiovascular mortality in middle age and elderly population.

Therefore, our study evaluated the association between the TyG index and all-cause and cause-specific mortality in middle age and elderly population based on a large representative database.

## Methods

### Study population

**Fig. 1:**
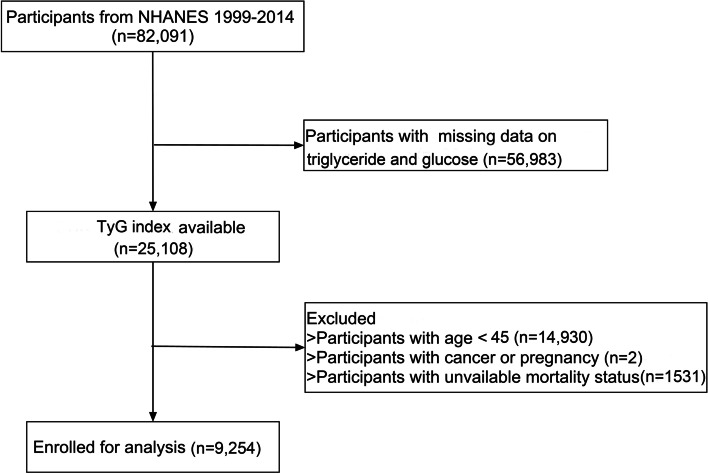
The flow chart of participant selection

The study included individuals from the National Health and Nutrition Examination Survey (NHANES) between the periods of 1999–2014, a nationwide survey conducted by the Centers for Disease Control and Prevention in United States. Sample weight were used to estimate the representative distribution using the supplied masked variance pseudo-stratum and masked variance pseudo-primary sampling units. Details of study implementation are available for online access (https://www.cdc.gov/nchs/nhanes/index.htm). Firstly, we excluded participants with missing data on triglyceride and glycose (*n* = 56,983) and participants with age < 45 years old (*n* = 14,930). In addition, we excluded those having cancer and pregnancy (*n* = 2), as well as unavailable mortality status (*n* = 1531). In total, 9,254 participants were enrolled in our study. Figure 1 depicted the selection process. Written informed consent was acquired from each participant and the protocol was approved by the Institutional Review Board of the Centers for Disease Control and Prevention.

### Exposure variable and outcomes

TyG index was calculated as ln [fasting triglycerides (mg/dL) x fasting glucose (mg/dL)/2] [14]. There was a median follow-up of 7.6 years. The primary outcome was all-cause mortality while the secondary outcomes included death from cardiovascular disease and malignant neoplasms. Mortality status was obtained by linkage to the National Death Index by 31 December 2015. Cardiovascular disease was defined as ICD-10 codes I00-I09, I11, I13, or I20-I51. Malignant neoplasm was defined as ICD-10 codes C00-C97 [15].

### Covariates collection

Information on age, sex, race, education level, poverty income ratio (PIR), smoking status, alcohol habit, activity habits, comorbid illness (hypertension [HBP], diabetes mellites [DM] and cardiovascular diseases [CVD], chronic obstructive pulmonary disease [COPD] and liver diseases) and medication use (hypoglycemic drug and lipid-lowering drug) were collected by using standardized questionnaires. Low density lipoprotein (LDL) and creatinine were measured by standard biochemistry assays. The height and weight of each participant were obtained from the physical examinations. Body mass index (BMI, kg/m2) was calculated as weight divided by height squared. Race/ethnicity was classified as non-Hispanic white, non-Hispanic black, Mexican American or other race. Education level was categorized as less than high school, high school or equivalent and college or above. PIR was categorized as < 1, 1–3, and > 3. Smoking status were defined as current, past and never. Activity habits was categorized as vigorous, moderate and inactive. The estimated glomerular filtration rate (eGFR) was calculated according to the Chronic Kidney Disease-Epidemiology Collaboration (CKD-EPI) equation. HBP was defined as the self-report hypertension, or systolic blood pressure ≥ 140 mmHg, or diastolic blood pressure ≥ 90 mmHg, or taking antihypertensive drugs. Diabetes was defined as a history of diabetes or fasting glucose > 7 mmol/L or glycated hemoglobin A1c > 6.5% or use of hypoglycemic medication. Multiple imputation was performed for covariates with missing values.

### Statistical analysis

Data are presented as mean ± SD or number (proportions). Differences among different TyG groups were explored by one-way analysis of variance and Chi-square test. Associations between TyG index and the risk of all-cause and specific-cause mortality were estimated by multivariate Cox regression models. The reference category f was the lowest TyG quartile. Model 1 was unadjusted. Model 2 was adjusted for age, gender, and race. Model 3 was adjusted for age, gender, race, education, PIR, BMI, smoker, drinking, physical activity, HBP, DM, CVD, hypoglycemic drug, lipid-lowering drug, LDL, and eGFR. The dose–response association was evaluated on a continuous scale with restricted cubic spline curves. If nonlinearity was detected, a two-piecewise Cox regression model was utilized to calculated the inflection point. All analysis were performed using R version 3.6. All P values were two-sided with a significance level of < 0.05.

## Results

The present study included 9,254 participants with a median 7.6-year-of follow-up. The baseline characteristics of the study population according to TyG quartile were shown in the Table 1. There were significant differences between TyG quartiles, except for drinking status and eGFR. Participants with a higher TyG index tended to have a higher BMI and more percentage of smokers, as well as more percentage of comorbidities. In addition, there were more presence of all-cause mortality and cardiovascular mortality in a higher TyG group.

**Table 1:** Characteristics of the study population

Variable	Q1 (*n* = 2306)	Q2 (*n* = 2330)	Q3 (*n* = 2318)	Q4 (*n* = 2300)	*P* value
Male (%)	1031 (44.7)	1154 (49.5)	1150 (49.6)	1249 (54.3)	< 0.001
Age, years	60.53 (11.39)	62.38 (11.54)	62.87 (11.13)	62.12 (10.66)	< 0.001
Race (%)					< 0.001
Non-Hispanic white	1059 (45.9)	1134 (48.7)	1159 (50.0)	1083 (47.1)	
Non-Hispanic black	728 (31.6)	464 (19.9)	348 (15.0)	289 (12.6)	
Mexican American	240 (10.4)	367 (15.8)	447 (19.3)	577 (25.1)	
Others	279 (12.1)	365 (15.7)	364 (15.7)	351 (15.3)	
Education (%)					< 0.001
Less than high school	634 (27.5)	738 (31.7)	802 (34.7)	919 (40.1)	
High school or equivalent	486 (21.1)	533 (22.9)	538 (23.3)	543 (23.7)	
College or above	1183 (51.4)	1054 (45.3)	971 (42.0)	831 (36.2)	
PIR (%)					< 0.001
< 1	332 (15.9)	377 (17.8)	379 (18.0)	439 (21.1)	
1 ~ 3	825 (39.6)	887 (41.8)	941 (44.6)	933 (44.9)	
> 3	928 (44.5)	859 (40.5)	791 (37.5)	708 (34.0)	
BMI, kg/m2	27.17 (6.27)	28.38 (6.07)	29.93 (6.25)	30.71 (5.95)	< 0.001
Drinking (%)	365 (54.5)	365 (51.3)	389 (52.4)	346 (50.3)	0.454
Smoking (%)					< 0.001
Current	344 (21.4)	389 (24.1)	352 (22.9)	409 (27.9)	
Past	53 (3.3)	40 (2.5)	67 (4.4)	65 (4.4)	
Never	1213 (75.3)	1185 (73.4)	1121 (72.8)	992 (67.7)	
Activity (%)					0.011
Vigorous	460 (36.4)	401 (33.0)	374 (32.3)	340 (30.8)	
Moderate	562 (44.5)	560 (46.1)	580 (50.1)	560 (50.7)	
Inactive	242 (19.1)	253 (20.8)	204 (17.6)	204 (18.5)	
Past history (%)					
HBP	567 (25.6)	617 (27.5)	702 (31.6)	742 (33.6)	< 0.001
DM	204 (8.8)	328 (14.1)	569 (24.5)	1140 (49.6)	< 0.001
CVD	219 (9.5)	283 (12.2)	317 (13.7)	394 (17.1)	< 0.001
COPD	129 (5.6)	144 (6.2)	151 (6.5)	192 (8.3)	0.001
Liver diseases	92 (4.0)	102 (4.4)	101 (4.4)	138 (6.0)	0.006
Prior medication (%)					
Hypoglycemic drug	109 (44.5)	183 (52.6)	293 (57.5)	539 (64.9)	< 0.001
Lipid-lowering drug	413 (86.6)	523 (82.4)	634 (83.0)	774 (81.0)	0.067
LDL, mg/dL	112.8 (32.2)	123.1 (35.2)	123.6 (37.1)	121.3 (42.1)	< 0.001
Glucose, mg/dL	96.8 (12.5)	103.7 (16.9)	110.9 (24.4)	144.4 (64.5)	< 0.001
Triglycerides, mg/dL	64.4 (15.1)	99.7 (16.9)	141.5 (27.5)	260.8 (183.7)	< 0.001
eGFR, ml/min per 1.73 m2	85.98 (25.03)	85.16 (24.67)	84.52 (26.40)	85.86 (29.12)	0.205
Mortality					
All-cause	371 (16.1)	437 (18.8)	450 (19.4)	516 (22.4)	< 0.001
Cardiovascular	68 (18.3)	62 (14.2)	87 (19.4)	113 (22.0)	0.021
Cancer	71 (19.1)	95 (21.7)	88 (19.6)	98 (19.1)	0.726

Data are presented as mean (SD) or n (%). Q1: TyG index ≤ 8.32; Q2: 8.32 ~ 8.72; Q3:8.72 ~ 9.16; Q4: > 9.16

*PIR* poverty income ratio, *BMI* body mass index, *HBP* high blood pressure, *DM* diabetes mellitus, *CVD* cardiovascular diseases, *COPD* chronic obstructive pulmonary diseases, *LDL* low density lipoprotein, *eGFR* estimated glomerular filtration rate

**Fig. 2:**
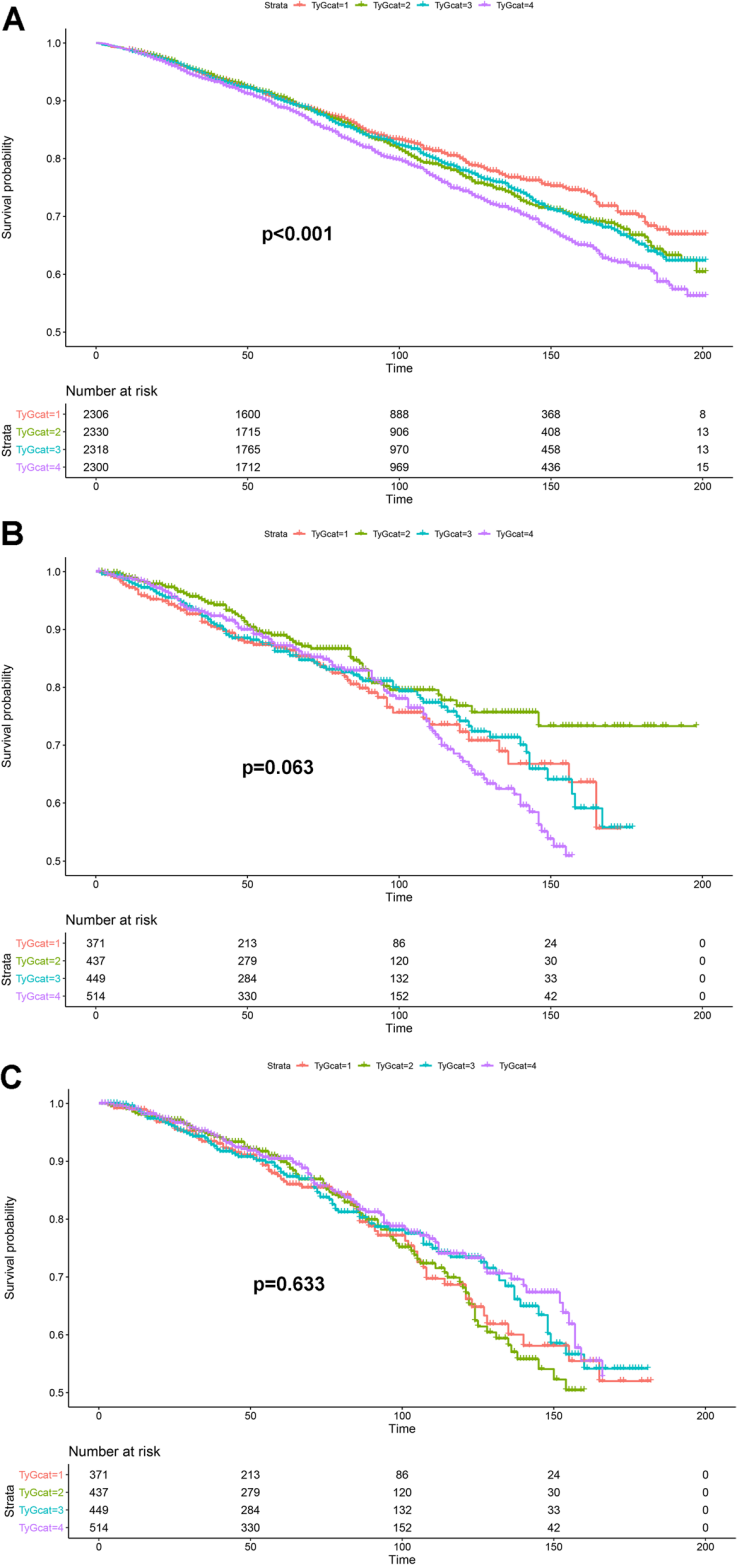
The Kaplan–Meier analysis of the prognostic effect of TyG index on all-cause mortality (**A**), cardiovascular mortality (**B**) and cancer mortality (**C**). TyGcat represented the quartiles of TyG index. The unit of time was Month

The Kaplan–Meier analysis was performed to explore the prognostic effect of TyG index on all-cause mortality (Fig. 2A), cardiovascular mortality (Fig. 2B) and cancer mortality (Fig. 2C). As shown, a higher TyG index was only associated with a higher risk of all-cause mortality (p for log-rank < 0.001).

As shown in Table 2, we constructed three models for analyzing the independent role of TyG index in mortality. When compared with the lowest quartile, the highest quartile of TyG increased the risk of all-cause mortality (HR 1.30, 95% CI [1.13, 1.48]; *p* < 0.001). Per 1-unit increasement of TyG was associated with 1.18-fold higher risk of mortality (HR 1.18, 95% CI [1.11, 1.26]; *p* < 0.001). However, after adjusted for all covariates, TyG index was not related to all-cause mortality. However, we observed a significant association between the third quartile of TyG index with all-cause mortality (HR 0.84, 95%CI [0.73, 0.98]; *p* < 0.05). In addition, a second quartile of TyG was associated with the lowest risk of cardiovascular mortality (HR 0.62, 95%CI [0.43, 0.88]; *p* < 0.01). However, no association between TyG quartile and cancer mortality was observed.

**Table 2:** Association of TyG with all-cause and cause specific mortality

All cause and cause specific mortality	Model 1 HR(95% CI)	Model 2 HR(95% CI)	Model 3 HR(95% CI)
All causes			
Q1	Ref	Ref	Ref
Q2	1.11 [0.97, 1.27]	0.95 [0.82, 1.09]	0.87 [0.75, 1.00]
Q3	1.11 [0.97, 1.27]	0.98 [0.85, 1.12]	0.84 [0.73, 0.98]*
Q4	1.30 [1.13, 1.48]***	1.25 [1.09, 1.44]**	0.90 [0.77, 1.04]
Continuous	1.18 [1.11, 1.26]***	1.23 [1.14, 1.32]***	1.01 [0.93, 1.10]
Cardiovascular			
Q1	Ref	Ref	Ref
Q2	0.69 [0.49, 0.98]*	0.70 [0.50, 0.99]*	0.62 [0.43, 0.88]**
Q3	0.94 [0.68, 1.29]	0.98 [0.71, 1.34]	0.82 [0.59, 1.15]
Q4	1.04 [0.77, 1.41]	1.18 [0.87, 1.61]	0.95 [0.67, 1.35]
Continuous	1.09 [0.93, 1.27]	1.18 [1.01, 1.39]*	1.03 [0.86, 1.25]
Malignant neoplasms			
Q1	Ref	Ref	Ref
Q2	1.00 [0.73, 1.36]	1.00 [0.73, 1.36]	1.08 [0.78, 1.49]
Q3	0.89 [0.65, 1.22]	0.92 [0.67, 1.26]	1.05 [0.75, 1.46]
Q4	0.85 [0.62, 1.15]	0.80 [0.59, 1.10]	0.94 [0.66, 1.34]
Continuous	0.88 [0.75, 1.03]	0.84 [0.72, 0.99]*	0.92 [0.76, 1.11]

Model 1 was unadjusted

Model 2 was adjusted for age, gender, and race

Model 3 was adjusted for age, gender, race, education, PIR, BMI, smoker, drinker, physical activity, HBP, DM, CVD, COPD, liver diseases hypoglycemic drug, lipid-lowering drug, LDL, eGFR

^*^p < 0.05, ^**^p < 0.01, ^***^p < 0.001; *HR*, hazard ratio, *CI*, confidence interval

**Fig. 3:**
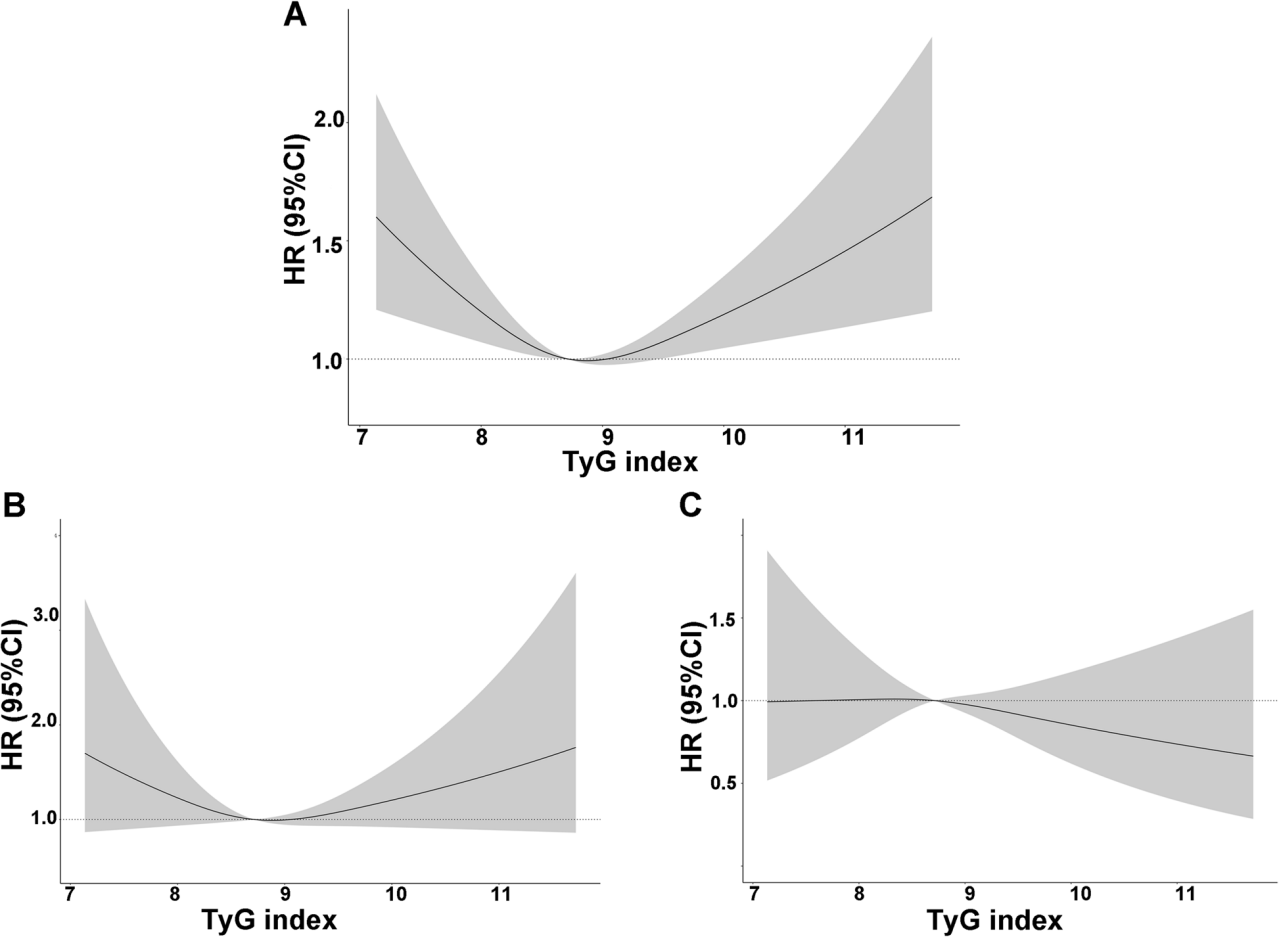
The restricted cubic regression between TyG index with all-cause mortality (**A**), cardiovascular mortality (**B**) and cancer mortality (**C**) in the fully adjusted model

**Fig. 4:**
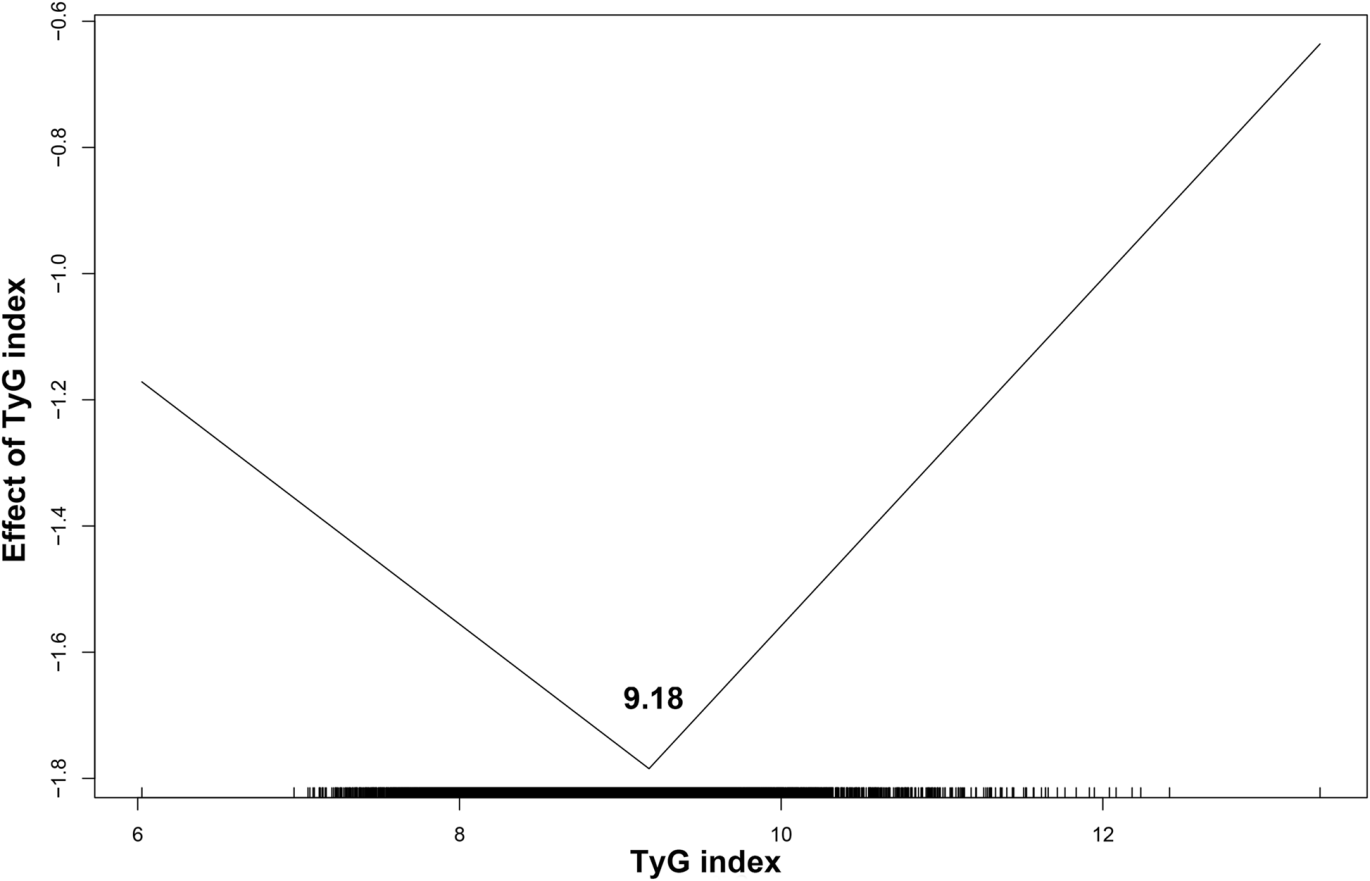
Threshold effect analysis of TyG on all-cause mortality using two piecewise Cox regression models

Restricted cubic spline regressions (Fig. 3A, B&C) suggested that TyG index was only nonlinearly associated with the risk of all-cause mortality (p for nonlinearity < 0.001). What’s more, we used two-piecewise Cox regression to determine the inflection point. As shown (Fig. 4& Table 3), the TyG index associated with the lowest risk of all-cause mortality was 9.18 and below 9.18, TyG index was negative associated with all-cause mortality (HR 0.82, 95%CI [0.71,0.96]) while above 9.18, TyG index was positively associated with all-cause mortality (HR 1.32, 95%CI [1.12,1.55]).

**Table 3:** Two-piecewise Cox regression analysis of the effect of TyG on all-cause mortality

TyG	Inflection point	Group	HR (95%CI)
Per 1-unit increasement	9.18	≤ 9.18	0.82 [0.71, 0.96]
		> 9.18	1.32 [1.12, 1.55]

## Discussion

In this study, we found TyG index was U-shaped associated with the risk of all-cause mortality after adjusting for traditional risk factors of mortality. And the TyG index with the lowest risk of all-cause mortality was 9.18 among middle age and elderly population. These results confirmed that TyG index could be an independent prognostic factor and have implications for the interpretation of levels of TyG in clinical practice.

TyG is usually associated with lipid and glucose metabolism disorders [16, 17], as a risk factor of cardiovascular diseases [18]. Many studies found that TyG index was an independent predictor for adverse cardiovascular events in both nondiabetic and diabetic subjects [19, 20]. Besides, based on a large cohort of older participants, Li et al. found that increased TyG index were significantly associated with an increased risk of cardiovascular diseases [21]. Our study found that TyG index ranging between 8.32 and 8.72 was associated with the lowest risk of cardiovascular mortality. These results suggested that keeping TyG under a propriate range was favorable for cardiovascular death.

A magnitude of publications reported that TyG increased the risk of all-cause mortality in patients with cardiovascular diseases and diabetes [7]. In general population [13] or hypertensive population [8], TyG index was non-linear associated with all-cause and cardiovascular mortality, and the threshold value was 9.36 for all-cause mortality. Even our univariate analysis found TyG was positively related to all-cause mortality, multivariable regression demonstrated that TyG was nonlinearly associated with the risk of mortality in general middle-age and elderly population, which was not affected by taking lipid-lowering drugs or hypoglycemic drugs. Specifically, when TyG was below 9.18, per 1-unit increasement decreased 0.82-fold risk of all-cause mortality, while TyG above 9.18, it was positively associated with the risk of all-cause mortality. This could be explained as follows. Firstly, low TG level was associated with the recurrent ischemia and the higher mortality of acute coronary syndrome[22]. Secondly, it was reported that higher TG had a potential protective role in vascular lesions. Thirdly, participants with high TyG index had more percentage of chronic illness, contributing to increased mortality. These results suggested that normalization of glucose or triglycerides under a target range was beneficial for a good prognosis.

Our study also has some limitations. Firstly, data on triglycerides and glucose were only collected once at baseline, and it was unclear whether TyG changes over time could affect the association with mortality. Secondly, the presence of chronic illness was self-reported based on questionnaires.

## Conclusions

In our study, we found TyG index was U-shaped associated with lower mortality in middle age and elderly population. And TyG index associated with the lowest risk of all-cause mortality was 9.18, which could be a prognostic factor in the clinical practice.

## Data Availability

The datasets generated and analyzed during the current study are not publicly available but are available from the corresponding author on reasonable request.
